# Review of the genus *Hieromantis* Meyrick from China, with descriptions of three new species (Lepidoptera, Stathmopodidae)

**DOI:** 10.3897/zookeys.534.5997

**Published:** 2015-11-11

**Authors:** Wei Guan, Houhun Li

**Affiliations:** 1 College of Life Sciences, Nankai University, Tianjin 300071, P. R. China

**Keywords:** Lepidoptera, Stathmopodidae, *Hieromantis*, new species, new record, China

## Abstract

Six species of the genus *Hieromantis* occurring in China are reviewed. *Hieromantis rectangula* **sp. n.**, *Hieromantis arcuata* **sp. n.** and *Hieromantis puerensis* **sp. n.** are described as new, and *Hieromantis phaeodora* Meyrick, 1929 is newly recorded in China. Photographs of adults and illustrations of genitalia are provided, along with an identification key and a distribution map.

## Introduction

The genus *Hieromantis* was established by Meyrick (1897) with *Hieromantis ephodophora* Meyrick, 1897 as its type species. It includes 17 named species worldwide: ten species were described from the Australian Region (Meyrick 1897, 1913, 1922, 1924, 1927, 1933, Bradley 1957), four from the Oriental Region (Meyrick 1907, 1913, 1929), one from the Palaearctic Region (Yasuda 1988), and two from both the Oriental and Palaearctic regions (Li and Wang 2002).

**Figure 1.**
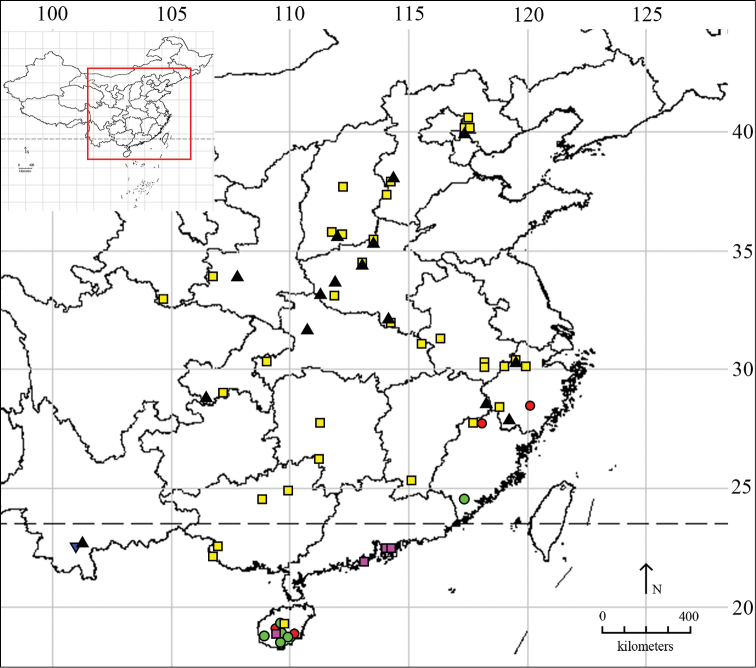
Distribution map of *Hieromantis* species in China. ● *Hieromantis rectangula* sp. n. ■ *Hieromantis arcuata* sp. n. ▼ *Hieromantis puerensis* sp. n. ● *Hieromantis phaeodora* Meyrick ■ *Hieromantis kurokoi* Yasuda ▲ *Hieromantis sheni* Li & Wang.

*Hieromantis kurokoi* Yasuda, 1988 and *Hieromantis sheni* Li & Wang, 2002 are the only species that were recorded in China prior to this study (Wang 2006). The aim of this paper is to review the *Hieromantis* species occurring in China. Three species are described as new to science, and one species is firstly recorded to China. The distribution map of *Hieromantis* species in China is shown in Fig. 1.

**Remarks.** The tube-shaped structure arising from the corpus bursae in the female genitalia is termed differently by different authors. It was described as a broad part of the corpus bursae plus the ductus seminalis by Kasy (1973), as ductus seminalis including the broad part and the narrow part by Yasuda (1988), and as the bulla plus the ductus seminalis by Koster and Sinev (2003). Here, we describe the structure between the corpus bursae and the ductus seminalis as the appendix bursae, and the abruptly narrowed distal portion as the ductus seminalis.

## Material and methods

The specimens examined in this study were collected using light traps. Morphological terminology follows Koster and Sinev (2003) except that the term appendix bursae is used instead of the term bulla. Genitalia dissections follow the methods introduced by Li (2002). Photographs of adults were taken with a Leica M205A Stereo microscope plus a Leica Application Suite 4.2 software, and photographs of genitalia were prepared using a Leica DM750 Microscope provided with the same software. All pictures were refined and assembled with Adobe Photoshop® CS6 software.

All the specimens, including the types, are deposited in the Insect Collection, College of Life Sciences, Nankai University, Tianjin, China.

## Taxonomy

### Hieromantis

Taxon classificationAnimaliaLepidopteraStathmopodidae

Meyrick, 1897

Hieromantis Meyrick, 1897: 315. Type species: *Hieromantis ephodophora* Meyrick, 1897, by monotypy.

#### Generic characters.

**Figures 2–4.**
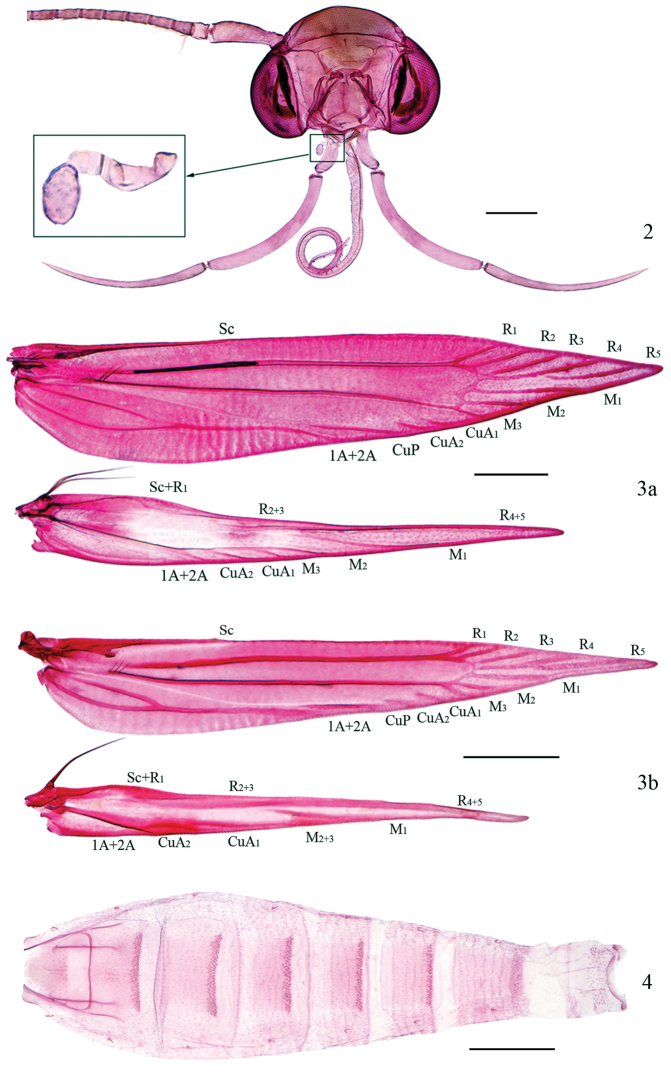
Morphological features of *Hieromantis* species. **2** *Hieromantis kurokoi* Yasuda, head, slide No. NKUGW004 **3a** Ditto, venation, slide No. GW13139W **3b** *Hieromantis rectangula* sp. n., venation, slide No. GW13143W **4** *Hieromantis kurokoi* Yasuda, abdomen (genitalia removed), slide No. NKUGW004. Scale bars: 0.2 mm (**2**); 0.5 mm (**3, 4**).

**Figures 5–12.**
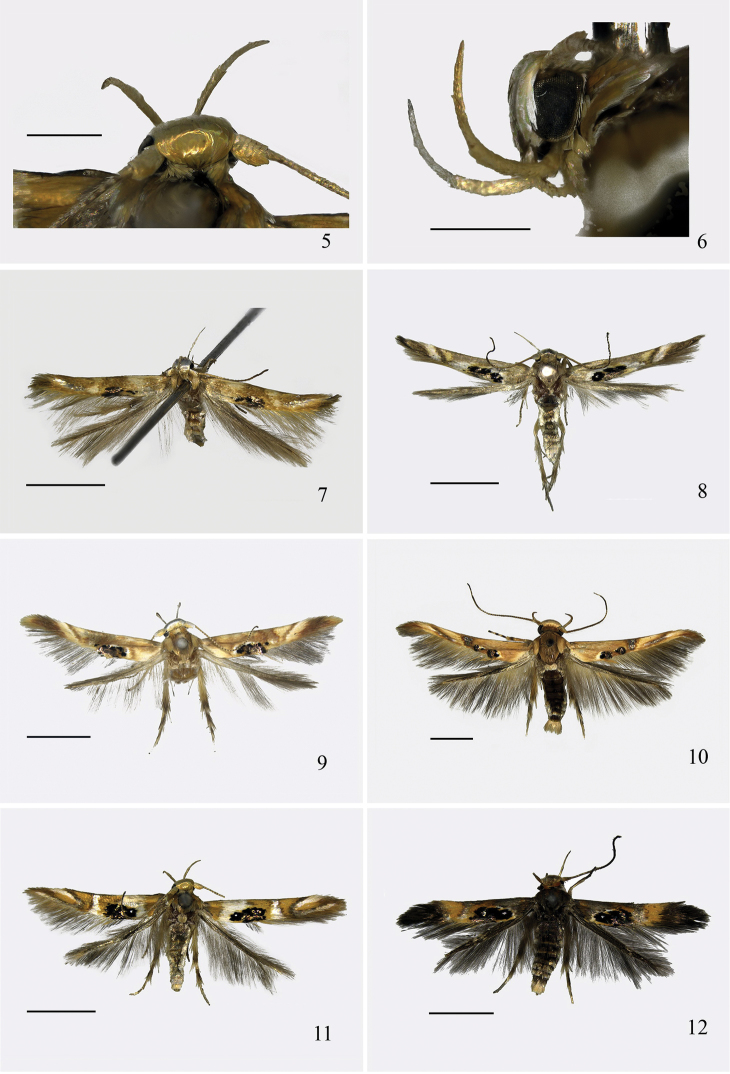
Adults of *Hieromantis* species. **5** *Hieromantis kurokoi* Yasuda, ♀, head (dorsal view) **6** Ditto, (lateral view) **7** *Hieromantis rectangula* sp. n., holotype, ♂ **8** *Hieromantis arcuata* sp. n., paratype, ♂ **9** *Hieromantis puerensis* sp. n., holotype, ♀ **10** *Hieromantis phaeodora* Meyrick, ♂ **11** *Hieromantis kurokoi* Yasuda, ♀ **12** *Hieromantis sheni* Li & Wang, ♀. Scale bars: 2.0 mm, except 0.5 mm (**5–6**).

**Adult.** Head smoothly scaled. Antenna (Figs 2, 5) with scape dilated, concave ventrally and forming an eye-cap; flagellum ciliated in male, simple in female. Labial palpus (Figs 2, 6) with third segment slightly longer than second segment. Maxillary palpus (Fig. 2) four-segmented, extremely short. Forewing lanceolate, usually having a loose patch consisting of scale tufts with metallic luster on dorsum; R_1_ and R_2_ arising from before upper angle of cell, R_3_ arising from upper angle of cell, R_4_ and R_5_ stalked, R_5_ reaching costa near apex; M_1_ and M_2_ nearly parallel, M_3_ arising from lower angle of cell; CuA_1_ and CuA_2_ very short, nearly parallel; 1A+2A furcate at base. Hindwing narrowly lanceolate, with long cilia approximately seven times width of wing; R_2+3_ reaching 2/5 of costa, R_4+5_ reaching costa before apex; M_2_ and M_3_ usually present (Fig. 3a), but sometimes coincident (Fig. 3b). Hindleg with tibia dorsally bearing tufts of erect scales, with bundles of bristles at apex. Abdominal tergites with second to seventh segments of male and second to sixth segments of female lined with spines on posterior margin (Fig. 4).

**Male genitalia.** Uncus usually subtriangular; gnathos broad tongue-shaped or subtriangular, about same length as uncus. Valva straight or obliquely upturned distally; sacculus weakly sclerotized. anellus lobes developed, usually longer than juxta. Aedeagus with a distal clubbed projection ventrally; cornutus present or absent.

**Female genitalia.** Intersegmental membrane between papilla analis and eighth abdominal segment usually longer than twice length of papilla analis. Antrum usually rectangular. Corpus bursae round or ovate, sometimes elongated; signum present or absent. Appendix bursae long tube shaped, usually with dilation. Ductus seminalis extremely thin.

#### Diagnosis.

The genus *Hieromantis* is related to *Stathmopoda* by sharing a ciliated antenna in male. It can be easily separated by the dilated scape that forms an eye-cap and the forewing that usually has a large patch with metallic luster on the dorsum.

#### Biology.

Little is known about the biology of *Hieromantis*. Larvae of *Hieromantis kurokoi* Yasuda, 1988 were found on *Cuscuta japonica* Choisy in autumn (Murase 2002).

#### Distribution.

China, Japan, Sri Lanka, the Andaman Islands, Russia, Australia, New Guinea, Samoa, Fiji, Guadalcanal Island.

#### Key to Chinese species of *Hieromantis*

**Table:** 

1	Male	**2**
−	Female	**6**
2	Cucullus rounded, not upturned (Fig. 16)	***Hieromantis kurokoi***
−	Cucullus subtriangular, usually upturned	**3**
3	Aedeagus without cornutus (Fig. 13)	***Hieromantis rectangula* sp. n.**
−	Aedeagus with cornutus	**4**
4	Uncus clubbed in distal 1/4 (Fig. 14)	***Hieromantis arcuata* sp. n.**
−	Uncus subrectangular in distal 1/4	**5**
5	Cornutus approximately 3/5 length of aedeagus, extending from basal 1/5 to distal 1/5 (Fig. 15)	***Hieromantis phaeodora***
−	Cornutus approximately 1/3 length of aedeagus, extending from basal 1/3 to distal 1/3 (Fig. 17)	***Hieromantis sheni***
6	Corpus bursae with signum	**7**
−	Corpus bursae without signum	**10**
7	Appendix bursae longer than four times length of corpus bursae	**8**
−	Appendix bursae about same length as corpus bursae	**9**
8	Intersegmental membrane between papilla analis and eighth abdominal segment longer than papilla analis, corpus bursae without rowed teeth (Fig. 19)	***Hieromantis arcuata* sp. n.**
−	Intersegmental membrane between papilla analis and eighth abdominal segment as long as papilla analis, corpus bursae with two parallel rows of teeth (Fig. 21)	***Hieromantis phaeodora***
9	Corpus bursae ovate, signum arcuate (Fig. 22)	***Hieromantis kurokoi***
−	Corpus bursae pyriform, signum linear, consisting of four basally joined teeth (Fig. 23)	***Hieromantis sheni***
10	Antrum subrectangular, without lamella antevaginalis (Fig. 18)	***Hieromantis rectangula* sp. n.**
−	Antrum indistinct, lamella antevaginalis consisting of a pair of kidney-shaped sclerites (Fig. 20)	***Hieromantis puerensis* sp. n.**

### Hieromantis rectangula sp. n.

Taxon classificationAnimaliaLepidopteraStathmopodidae

http://zoobank.org/6AC5AEDE-2E26-4F50-A3BC-0BAA703420FF

Figs 7 , 13 , 18

#### Type material.

**CHINA: Holotype** ♂, Mt. Diaoluo (18°28'N, 109°31'E), Hainan Province, 940 m, 31.v.2007, leg. Zhiwei Zhang & Weichun Li, genitalia slide No. GW13134. **Paratypes: Hainan Province**: 4♂, Mt. Wuzhi, 700 m, 19.v.2007, leg. Zhiwei Zhang & Weichun Li; 5♂, 6♀, Mt. Wuzhi, 742 m, 18−22.v.2015, leg. Peixin Cong, Wei Guan & Sha Hu; 1♂, 2♀, Jianfengling, 940 m, 4−5.vi.2007, leg. Zhiwei Zhang & Weichun Li; 1♀, Jianfengling, 810 m, 30.iii.2008, leg. Bingbing Hu & Haiyan Bai; 1♂, Jianfengling, 770 m, 16.vii.2014, leg. Peixin Cong, Linjie Liu & Sha Hu; 10♂, 13♀, Jianfengling, 770 m, 28.v.−5.vi.2015, leg. Peixin Cong, Wei Guan & Sha Hu; 1♀, Mt. Limu, 607 m, 16.v.2015, leg. Peixin Cong, Wei Guan & Sha Hu; **Zhejiang Province**: 1♂, Mt. Jiulong, 400 m, 6.viii.2011, leg. Linlin Yang & Na Chen; **Fujian Province**: 1♀, Mt. Wuyi, 740 m, 17.v.2004, leg. Haili Yu.

#### Diagnosis.

This new species is similar to *Hieromantis makiosana* Yasuda, 1988 by the similar forewing markings, but can be distinguished by the inverted triangular costal patch on the forewing having a black dot at its ventral angle; the cucullus slightly concave on the outer margin near the junction with the ventral margin of the valva and the rectangular juxta in the male genitalia; and the ostium without minute spines in the female genitalia. In *Hieromantis makiosana*, the similar costal patch on the forewing lacks a black dot at its ventral angle; the cucullus is straight on the outer margin and the juxta is ovate; and the ostium has minute spines.

#### Description.

Adult (Fig. 7). Wingspan 6.0−8.5 mm. Head with frons silvery white; vertex mottled with ochreous yellow scales; occiput yellowish brown. Labial palpus ochreous yellow on outer surface, silvery white on inner surface. Antenna with scape pale yellowish brown, edged with snowy white scales anteriorly; flagellum ochreous yellow, with brown rings. Thorax and tegula pale yellowish brown. Forewing creamy white, with scattered yellowish brown scales, ochreous yellow from dorsal 2/3 along dorsum to apex; trapezoidal ochreous yellow patch extending from between costal 1/3 and 2/5 to between dorsal 1/4 and 1/2; inverted triangular ochreous yellow patch extending from between costal 3/5 and 4/5 to lower angle of cell, bearing an ill-defined black dot posteriorly, with a narrow silvery gray band placed along its outer margin; dorsum with an ovate dark blotch located between basal 1/4 and 2/5 consisting of tufts of shining purple gray scales, its anterior margin cross 2/5 width of wing, near its inner side set a black spot; cilia pale yellowish brown. Hindwing grayish brown; cilia yellowish brown. Legs pale yellowish brown: foreleg with tibia purple brown on inner side, yellowish white on outer side, tarsus ringed with dark brown scales at apices of basal two tarsomeres; mid tibia dorsally with long yellowish brown bristles at middle and at apex, tarsus ringed with blackish brown scales at apices of basal two tarsomeres; hind tibia dorsally with dense pale grayish yellow bristles, ringed with blackish brown bristles at apex, tarsus dorsally with pale grayish yellow bristles on basal two tarsomeres, ringed with blackish brown bristles at apices of basal three tarsomeres. Abdomen ochreous gray on dorsal surface, shining white on ventral surface; lateral sides and anal tuft grayish white.

**Figures 13–17.**
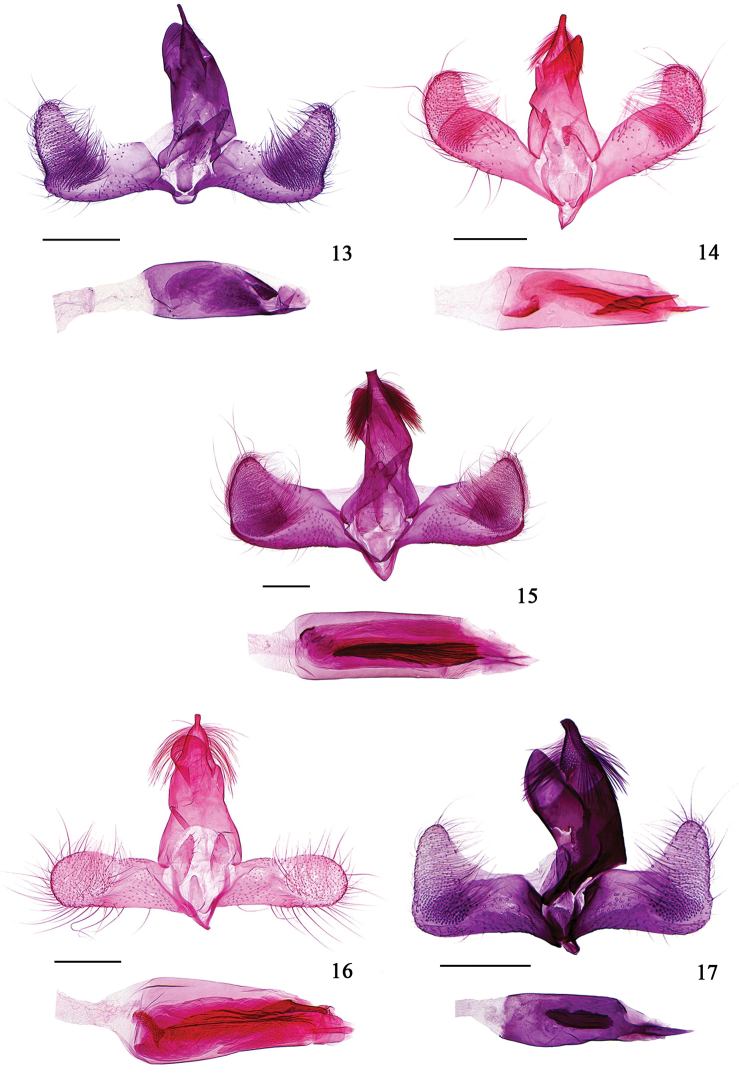
Male genitalia of *Hieromantis* species. **13** *Hieromantis rectangula* sp. n., holotype, slide No. GW13134 **14** *Hieromantis arcuata* sp. n., holotype, slide No. GW13203 **15** *Hieromantis phaeodora* Meyrick, slide No. GW14027 **16** *Hieromantis kurokoi* Yasuda, slide No. GW13138 **17** *Hieromantis sheni* Li & Wang, slide No. GW12299. Scale bars: 0.2 mm.

**Male genitalia** (Fig. 13). Uncus basally broad, gradually narrowed to 2/3, with sparse long hairs laterally, distal 1/3 thin clubbed. Gnathos broad tongue-shaped, bluntly rounded at apex. Tegumen about 1.3 times length of uncus. Valva narrow basally, broadened distally; costa concave at middle; sacculus narrow, slightly concave near base on ventral margin, then convex up to junction with cucullus; cucullus elongate triangular, obliquely upward-oriented, narrowly rounded at apex, slightly concave on outer margin near junction with ventral margin of valva. Vinculum narrowly banded; saccus short rectangular, about 1/6 length of uncus. Juxta rectangular, anterior margin sclerotized, pointed medially, posterior margin bluntly rounded; anellus lobes elongate clubbed, about twice length of juxta. Aedeagus about 1.2 times length of valva, basal 2/3 nearly uniform, distal 1/3 gradually narrowed, produced to a thin clubbed distal projection ventrally, sclerotized near apex dorsally; with numerous tiny spines extending from basal 1/2 to 5/6; cornutus absent.

**Figures 18–21.**
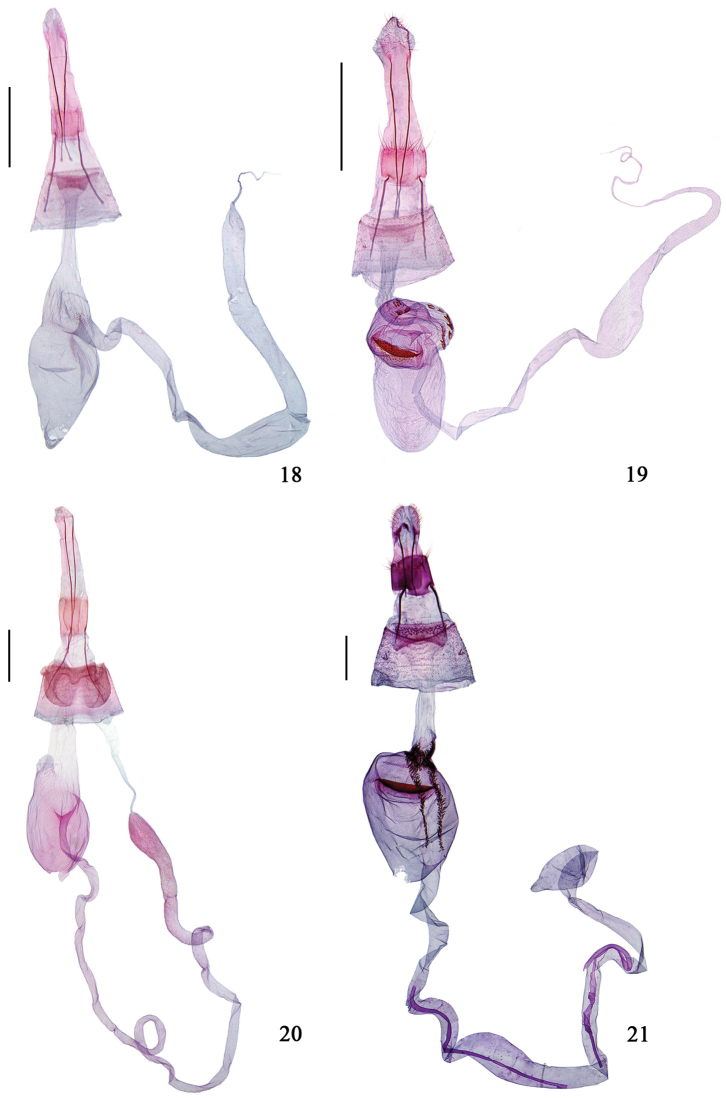
Female genitalia of *Hieromantis* species. **18** *Hieromantis rectangula* sp. n., paratype, slide No. GW13144 **19** *Hieromantis arcuata* sp. n., paratype, slide No. GW13146 **20** *Hieromantis puerensis* sp. n., holotype, slide No. GW13142 **21** *Hieromantis phaeodora* Meyrick, slide No. GW12266. Scale bars: 0.5 mm.

**Female genitalia** (Fig. 18). Intersegmental membrane between papilla analis and eighth abdominal segment about three times length of papilla analis. Apophysis posterior about 1.5 times length of apophysis anterior. Eighth segment with both anterior and posterior margins straight, with long hairs on posterior margin. Antrum subrectangular, slightly concave on both anterior and posterior margins, width about four times length. Ductus bursae uniformly narrow in posterior 3/4, gradually widened anteriorly, not distinctly separated from corpus bursae. Corpus bursae nearly fusiform, with dense granules near junction with appendix bursae; signum absent. Appendix bursae arising from junction between corpus bursae and ductus bursae, broad tube shaped, longer than four times length of corpus bursae, with several small teeth at base, slightly dilated in medial section.

#### Distribution.

China (Fujian, Hainan, Zhejiang).

#### Etymology.

The specific name is derived from the Latin *rectangulus*, referring to the rectangular juxta in the male genitalia.

### Hieromantis arcuata sp. n.

Taxon classificationAnimaliaLepidopteraStathmopodidae

http://zoobank.org/45E1C0A4-47E7-4044-BD44-2BE214C7D881

Figs 8 , 14 , 19

#### Type material.

**CHINA: Holotype** ♂, Jiadaoli Farm (22°26'N, 114°07'E), Hong Kong, 210 m, 12.iv.2007, leg. Houhun Li *et al*., genitalia slide No. GW13203. **Paratypes: Hong Kong**: 1♀, same data as holotype; 1♂, same data as holotype except dated 10.iv.2007; 1♂, 340−455 m, 14.iv.2007, other data same as holotype; 2♀, Gaotang, Xigong, 100 m, 25.ix.2009, leg. Houhun Li; 1♂, Kadoorie Agric. Reaearch Centre Shek Kong N. T., 200 m, 5.viii.1998, leg. R. C. Kendrick; **Guangdong Province**: 1♂, Hebao Island, Zhuhai City, 8 m, 19.i.2010, leg. Houhun Li; **Hainan Province**: 1♀, Mt. Diaoluo, 940 m, 1.vi.2007, leg. Zhiwei Zhang & Weichun Li; 1♀, Mt. Wuzhi, 740 m, 14.iv.2009, leg. Qing Jin & Bingbing Hu; 1♀, Yinggeling, 620 m, 21.v.2010, leg. Bingbing Hu & Jing Zhang; 1♀, Yinggeling, 508 m, 15.vi.2015, leg. Peixin Cong, Wei Guan & Sha Hu; 2♀, Jianfengling, 770 m, 14−16.vii.2014, leg. Peixin Cong, Linjie Liu & Sha Hu; 1♀, Jianfengling, 770 m, 30.v.2015, leg. Peixin Cong, Wei Guan & Sha Hu.

#### Diagnosis.

This new species is characterized by the valva having a straight wrinkle that extends from 3/4 of the costa directly to the ventral margin and the costa arched in basal 3/4 in the male genitalia. It is similar to *Hieromantis kurokoi* Yasuda, 1988 by having similar forewing markings, but can be distinguished by having a subtriangular cucullus in the male genitalia; and the signum surrounded by dense small teeth and the appendix bursae longer than four times length of the corpus bursae in the female genitalia. In *Hieromantis kurokoi*, the cucullus is rounded; there are no small teeth surrounding the signum, and the appendix bursae is approximately as long as the corpus bursae.

#### Description.

Adult (Fig. 8). Wingspan 8.0−9.0 mm. Head with frons silvery white; vertex pale yellowish brown, with suffused gray scales; occiput grayish brown. Labial palpus pale yellowish brown, second segment silvery white on inner side, third segment pale brown on outer side. Antenna with scape pale yellowish brown, margined with yellowish white scales; flagellum brown. Thorax grayish brown; tegula grayish brown in anterior half, silvery gray in posterior half. Forewing grayish brown, with scattered yellowish brown scales, distal 1/5 ochreous brown; grayish brown band extending from costal 2/3 obliquely inward to dorsal 3/5, its inner side set an ill-defined ochre-yellow patch neither reaching costa nor dorsum, its outer side placed a broad silvery white band; dorsum with an elliptical spot located between basal 1/4 and 2/5 consisting of tufts of erect silvery gray scales with metallic luster, its inner side placed a rounded black spot with a white dot in centre, anterior to it set two joined black spots along middle of fold, these four spots forming a large elliptical blotch located between dorsal 1/5 and 2/5, margined with silvery white scales except on dorsum and yellowish brown in middle of cell; cilia pale brown. Hindwing and cilia grayish brown. Legs pale yellowish: fore tibia yellowish brown, tarsus brown; mid tibia dorsally with yellowish white scales in basal half and at apex, ringed with pale brown bristles at apex, tarsus ringed with pale brown bristles at apices of basal two tarsomeres; hind tibia dorsally with dense pale gray deepening to grayish brown bristles, with bundles of pale gray bristles at apex, tarsus dorsally with grayish brown scales on first tarsomere, with brassy brown bristles at apices of basal three tarsomeres, black distally. Abdomen dorsally grayish brown; ventrally grayish white; lateral sides and anal tuft pale gray.

**Male genitalia** (Fig. 14). Uncus basally broad, gradually narrowed to 3/4, with long hairs laterally; distal 1/4 short clubbed, bluntly rounded at apex. Gnathos tongue-shaped, with small granules at apex. Tegumen approximately 1.3 times length of uncus. Valva narrow at base, medial portion with dorsal and ventral margins subparallel; with a straight wrinkle extending from 3/4 of costa to ventral margin; costa arched in basal 3/4, concave at 3/4; sacculus straight, apically reaching outer margin of cucullus; cucullus subtriangular, obliquely upturned, rounded at apex, bluntly rounded on outer margin. Vinculum narrowly banded; saccus V shaped, about 1/3 length of uncus. Juxta subrounded, anterior margin sclerotized, with a small pointed medial process; anellus lobes long clubbed, about 2.5 times length of juxta. Aedeagus approximately 1.2 times length of valva, basal 4/5 about uniform, distal 1/5 gradually narrowed, produced to a distal clubbed projection ventrally; with a sclerotized plate placed near base; cornuti consisting of a bunch of fine sclerites extending from basal 1/4 to distal 1/5, and a thick thorn-shaped spine placed between basal 3/5 and near apex centrally.

**Female genitalia** (Fig. 19). Intersegmental membrane between papilla analis and eighth abdominal segment about twice length of papilla analis. Apophysis posterior about 1.5 times length of apophysis anterior. Eighth segment straight on posterior margin, with long hairs; eighth tergite with anterior 1/4 rectangular; eighth sternite straight on anterior margin. Antrum subrectangular, width about three times length, concave on anterior margin. Ductus bursae about 3/4 length of corpus bursae, uniformly narrow in posterior 5/6, gradually widened anteriorly. Corpus bursae ovate, with numerous granules posteriorly; signum crescent, arched on anterior edge, dentate on posterior edge, surrounded by dense small teeth. Appendix bursae arising from junction between corpus bursae and ductus bursae, longer than four times length of corpus bursae, lined with several large teeth at base, dilated elliptically between basal 1/2 and 2/3.

#### Distribution.

China (Guangdong, Hainan, Hong Kong).

#### Etymology.

The specific name is derived from the Latin *arcuatus*, referring to the costa arched in the basal 3/4 in the male genitalia.

### Hieromantis puerensis sp. n.

Taxon classificationAnimaliaLepidopteraStathmopodidae

http://zoobank.org/6E3850E2-699A-446C-B7B4-391AC7A4A216

Figs 9 , 20

#### Type material.

**CHINA: Holotype** ♀, Taiyanghe National Forest Park (22°36'N, 101°07'E), Pu’er City, Yunnan Province, 1626 m, 7.vii.2013, leg. Shurong Liu, Yuqi Wang & Kaijian Teng, genitalia slide No. GW13142. **Paratypes**: 2♀, same data as holotype except dated 6−7.vii.2013.

#### Diagnosis.

This species is superficially similar to *Hieromantis ephodophora* Meyrick, 1897, *Hieromantis phaeodora* Meyrick, 1929, *Hieromantis kurokoi* Yasuda, 1988 and *Hieromantis arcuata* sp. n. It can be easily distinguished from its allies by the female genitalia having a lamella antevaginalis that consists of a pair of posteriorly joined kidney-shaped sclerites and the absence of a signum.

#### Description.

Adult (Fig. 9). Wingspan 9.0−10.5 mm. Head with frons shining white; vertex pale yellowish brown; occiput pale ochreous yellow. Labial palpus silvery white, second segment with scattered pale yellow scales on outer side, third segment yellowish brown on outer side. Antenna with scape silvery white, with suffused yellowish brown scales posteriorly; flagellum ochreous yellow, ringed with brown. Thorax ochreous brown, with a longitudinal silvery white band in anterior half medially; tegula grayish yellow. Forewing brown, distal 1/5 ochreous brown; below costal margin set three pale ochreous yellow shades in basal half; broad grayish brown band extending from costal 1/2 slightly obliquely outward to above distal end of fold, ending in a silvery white spot, edged with narrow ochreous brown band along its inner and outer margins, with a rounded black dot placed at end of inner band above fold, its inner side with a few silvery white scales; inverted trapezoidal pale ochreous yellow patch located between outer band and costal 4/5, its posterior margin reaching beyond end of fold; narrow ochreous brown band extending from costal 3/4 obliquely inward to beyond end of fold, edged with scattered silvery white scales along outer side; dorsum with an ill-defined yellowish white patch at base, with a subovate patch located between basal 1/3 and before middle consisting of tufts of erect shining gray scales, surrounded by six not well separated black spots, margined with white scales along inner and anterior margins; cilia pale brown. Hindwing grayish brown, cilia pale brown. Legs pale yellow: foreleg ringed with black scales at apices of tibia and second tarsomere; mid tibia dorsally with yellowish white hairs, with white bristles at apex, tarsus ringed with blackish brown bristles at apex of second tarsomere; hind tibia dorsally with yellowish brown deepening to brown bristles, tarsus dorsally with grayish brown deepening to brown bristles on basal two tarsomeres, ringed with brown bristles at apices of basal two tarsomeres, distal two tarsomeres snowy white, black at apex. Abdomen dorsally ochreous brown; ventrally pale grayish white.

**Female genitalia** (Fig. 20). Intersegmental membrane between papilla analis and eighth abdominal segment about 3.5 times length of papilla analis. Apophysis posterior 1.5 times length of apophysis anterior. Eighth segment straight on both anterior and posterior margins, with long hairs on posterior margin. Lamella antevaginalis consisting of a pair of posteriorly joined kidney-shaped sclerites. Antrum indistinct. Ductus bursae approximately 1.3 times length of corpus bursae, narrow in posterior 1/4, slightly broadened anteriorly. Corpus bursae ovate, with dense granules entirely, with a protrudence carrying dense granules at junction with ductus bursae; signum absent. Appendix bursae arising from junction between corpus bursae and ductus bursae, long, tube shaped, longer than five times length of corpus bursae, slightly dilated near transition with ductus seminalis.

**Male.** Unknown.

#### Distribution.

China (Yunnan).

#### Etymology.

The specific name is derived from the type locality.

### Hieromantis phaeodora

Taxon classificationAnimaliaLepidopteraStathmopodidae

Meyrick, 1929

Figs 10 , 15 , 21

Hieromantis phaeodora Meyrick, 1929: 541. Type locality: Andaman Islands.

#### Material examined.

**CHINA: Fujian Province**: 1♀, Hexi Town, Nanjing County, Zhangzhou City, 295 m, 29.v.2011, leg. Jin Zhang; **Hainan Province**: 2♀, Jianfengling, 940 m, 5.vi.2007, leg. Zhiwei Zhang & Weichun Li; 1♂, 1♀, Tianchi, Jianfengling, 1050 m, 29−30.iv.2013, leg. Yinghui Sun, Wei Guan & Tengteng Liu; 1♂, Jianfengling, 1050 m, 27.iv.2014, leg. Tengteng Liu, Wei Guan & Xuemei Hu; 1♂, Jianfengling, 770 m, 14.vii.2014, leg. Peixin Cong, Linjie Liu & Sha Hu; 2♂, 1♀, Mt. Limu, 700 m, 13.iv.2008, leg. Bingbing Hu & Haiyan Bai; 17♂, 17♀, Mt. Limu, 640 m, 1.v.2014, leg. Tengteng Liu, Wei Guan & Xuemei Hu; 1♂, Nanxi Protection Station, Mt. Diaoluo, 250 m, 22.iv.2008, leg. Bingbing Hu & Haiyan Bai; 4♂, 2♀, Mt. Diaoluo, 980 m, 23−24.iv.2014, leg. Tengteng Liu, Wei Guan & Xuemei Hu; 2♂, Hongxin Village, Yuanmen Town, Baisha County, 430 m, 15−18.iv.2014, leg. Tengteng Liu, Wei Guan & Xuemei Hu; 1♂, Shuiman Town, Wuzhishan City, 620 m, 19.iv.2014, leg. Tengteng Liu, Wei Guan & Xuemei Hu; 2♂, 6♀, Mt. Wuzhi, 710 m, 21.iv.2014, leg. Tengteng Liu, Wei Guan & Xuemei Hu; 1♀, Mt. Wuzhi, 742 m, 20.v.2015, leg. Peixin Cong, Wei Guan & Sha Hu.

#### Diagnosis.

Adult (Fig. 10) with wingspan 14.0−15.0 mm. This species is characterized by the forewing having two separated dorsal spots that consist of tufts of erect purple gray scales; and the appendix bursae arising from the anterior 1/5 of the corpus bursae in the female genitalia. It is similar to *Hieromantis sheni* Li & Wang, 2002 by the obliquely upward-oriented triangular cucullus, but *Hieromantis phaeodora* can be distinguished from the latter by the foliaceous anellus lobes, the aedeagus about 1.8 times length of the valva and the cornutus about 3/5 length of the aedeagus in the male genitalia (Fig. 15); the corpus bursae having two parallel rows of teeth in the female genitalia (Fig. 21). In *Hieromantis sheni*, the anellus lobes are long clubbed, the aedeagus is almost as long as the valva, and the cornutus is about 1/3 length of the aedeagus; and the corpus bursae lacks two rows of teeth.

#### Distribution.

China (Fujian, Hainan), the Andaman Islands.

#### Notes.

This species is recorded for the first time in China.

### Hieromantis kurokoi

Taxon classificationAnimaliaLepidopteraStathmopodidae

Yasuda, 1988

Figs 11 , 16 , 22

Hieromantis kurokoi Yasuda, 1988: 494. Type locality: Japan.
Hieromantis nordella Sinev, 1988: 109.

#### Material examined.

**CHINA: Tianjin**: 1♂, Heishuihe, Mt. Baxian, Ji County, 550 m, 10.vi.2009, leg. Bingbing Hu; 1♀, Heishuihe, Mt. Baxian, Ji County, 600 m, 29.viii.2010, leg. Yinghui Mou & Shurong Liu; 1♀, Xiaogang, Ji County, 29.vi.2013, leg. Tengteng Liu; **Hebei Province**: 1♀, Laoniuyu Village, Jingxing County, Shijiazhuang City, 26.vii.2000, leg. Haili Yu; 1♂, Suanzaoping Village, Neiqiu County, Xingtai City, 670 m, 29.vii.2000, leg. Haili Yu; 1♀, Mt. Wuling, Xinglong County, 800 m, 27.vii.2011, leg. Houhun Li & Yanpeng Cai; **Shanxi Province**: 1♀, Dahe Forest Farm, 1340 m, 15.vii.2012, leg. Qiang Gao & Na Chen; 1♀, Dashuang Village, Magedang Town, Lingchuan County, 773 m, 21.vii.2012, leg. Wei Guan & Xiuchun Wang; 1♂, Zhangma Village, Mt. Li Nature Reserves, Qinshui County, Jincheng City (collected using sweep net in the day), 20.vii.2013, leg. Mingjing Li; 1♀, Dahe Forest Farm, Yicheng County, Linfen City, 1202 m, 24.vii.2013, leg. Shulian Hao & Mingjing Li; 1♀, Sijiao Forest Farm, Taikuanhe Nature Reserves, Linfen City, 893 m, 6.viii.2013, leg. Shulian Hao & Yunfei Peng; 1♂, 2♀, Taikuanhe Nature Reserves, Linfen City, 1020 m, 7.viii.2013, leg. Shulian Hao & Yunfei Peng; 3♀, Taikuanhe Nature Reserves, Linfen City, 1020 m, 8.viii.2013, leg. Shulian Hao & Mingjing Li; 1♂, 3♀, Mt. Tianlong, Taiyuan City, 1280 m, 14−15.vii.2013, leg. Tengteng Liu & Peixin Cong; **Zhejiang Province**: 1♂, 1♀, Chanyuan Temple, Mt. Tianmu, 350 m, 15.v.1999, leg. Houhun Li; 1♀, Tianmu Village, Mt. Tianmu, 335 m, 1.vii.2014, leg. Aihui Yin, Xuemei Hu & Qingyun Wang; 1♀, Laoan, Mt. Tianmu, 555 m, 3.vii.2014, leg. Aihui Yin, Xuemei Hu & Qingyun Wang; 1♀, Xiguan, Mt. Tianmu, 566 m, 19.vii.2014, leg. Aihui Yin, Xuemei Hu & Qingyun Wang; 1♀, Neijiuxian Village, Mt. Jiulong, 430 m, 7.vii.2013, leg. Aihui Yin & Xiuchun Wang; 2♀, Yulingguan, Qingliang Peak, 220 m, 23.vii.2014, leg. Aihui Yin, Xuemei Hu & Qingyun Wang; 2♀, Jiufu Village, Mt. Longtang, 520 m, 26.vii.2014, leg. Aihui Yin, Xuemei Hu & Qingyun Wang; 4♀, Jiufu Village, Mt. Longtang, 520 m, 27−31.viii.2014, leg. Aihui Yin, Qingyun Wang & Suran Li; 1♀, Longxushan Village, Mt. Longxu, 778 m, 22.viii.2014, leg. Aihui Yin, Qingyun Wang & Suran Li; 1♂, Pinggang, Mt. Longxu, 754 m, 23.viii.2014, leg. Aihui Yin, Qingyun Wang & Suran Li; **Anhui Province**: 2♂, 1♀, Tangkou Town, Huangshan City, 5.viii.2004, leg. Jiasheng Xu & Jialiang Zhang; 2♂, 4♀, Tanqiao Town, Huangshan City, 6−7.viii.2004, leg. Jiasheng Xu & Jialiang Zhang; 4♀, Mozitan Town, Huoshan County, 12.viii.2004, leg. Jiasheng Xu & Jialiang Zhang; **Fujian Province**: 2♀, San’gang, Mt. Wuyi, 740 m, 24.v.2004, leg. Haili Yu; **Jiangxi Province**: 1♀, Mt. Jinpen, 18.vii.2006, leg. Jiasheng Xu & Weichun Li; **Henan Province**: 1♂, 1♀, Lingshan Temple, Luoshan County, Xinyang City, 350 m, 23.v.1999, leg. Haili Yu; 2♀, Mt. Song, Dengfeng City, 800 m, 9.vi.2000, leg. Haili Yu; 1♀, Mt. Baotianman, Neixiang County, 1200 m, 13.viii.2006, leg. Hui Zhen & Denghui Kuang; 1♂, Zhuyu Peak, Mt. Yuntai, Jiaozuo City, 1297 m, 11.viii.2014, leg. Peixin Cong, Sha Hu & Linjie Liu; 1♀, Xiuwu Forest Farm, Mt. Yuntai, Jiaozuo City, 1028 m, 14.viii.2014, leg. Peixin Cong, Sha Hu & Linjie Liu; **Hubei Province**: 2♀, Maoba Region, Lichuan City, 700 m, 30.vii.1999, leg. Hounhun Li *et al.*; 1♀, Qingtaiguan Forest Farm, Luotian County, 570 m, 3.vii.2014, leg. Wei Guan & Meiqing Yang; **Hunan Province**: 1♂, 1♀, Cangxi Town, Jinhua County, 8−9.viii.2004, leg. Yunli Xiao; **Guangxi Zhuang Autonomous Region**: 1♀, Qinmu Village, Yongfu County, 160 m, 1.v.2008, leg. Hui Zhen & Li Zhang; 2♀, Qingshan Forest Farm, Pingxiang City, 300 m, 21.vii.2011, leg. Bingbing Hu *et al.*; 1♂, Zhoutong Village, Nanping Town, Yizhou City, 450 m, 17.viii.2011, leg. Shulian Hao & Yinghui Sun; 2♂, 6♀, Shaoping Forest Farm, Pingxiang City, 280 m, 28.iii.2012−19.iv.2012, leg. Xiaofei Yang; 1♀, Nonggang Protection Station, Longzhou County, 25.vii.2013, leg. Xiaofei Yang; 2♀, Miaotou Town, Quanzhou County, Guilin City, 100 m, 26.vii.2013, leg. Xiaofei Yang; **Hainan Province**: 1♀, Mt. Limu, Qiongzhou County, 640 m, 2.v.2014, leg. Tengteng Liu, Wei Guan & Xuemei Hu; **Chongqing**: 1♀, Mt. Jinfo, 1100 m, 6.vi.2013, leg. Xiaofei Yang; **Shaanxi Province**: 1♀, Shuangshipu Town, Feng County, 1050 m, 23.viii.1987, leg. Houhun Li; **Gansu Province**: 1♀, Bifenggou, Wen County, 860 m, 9.vii.2005, leg. Haili Yu.

**Figures 22–23.**
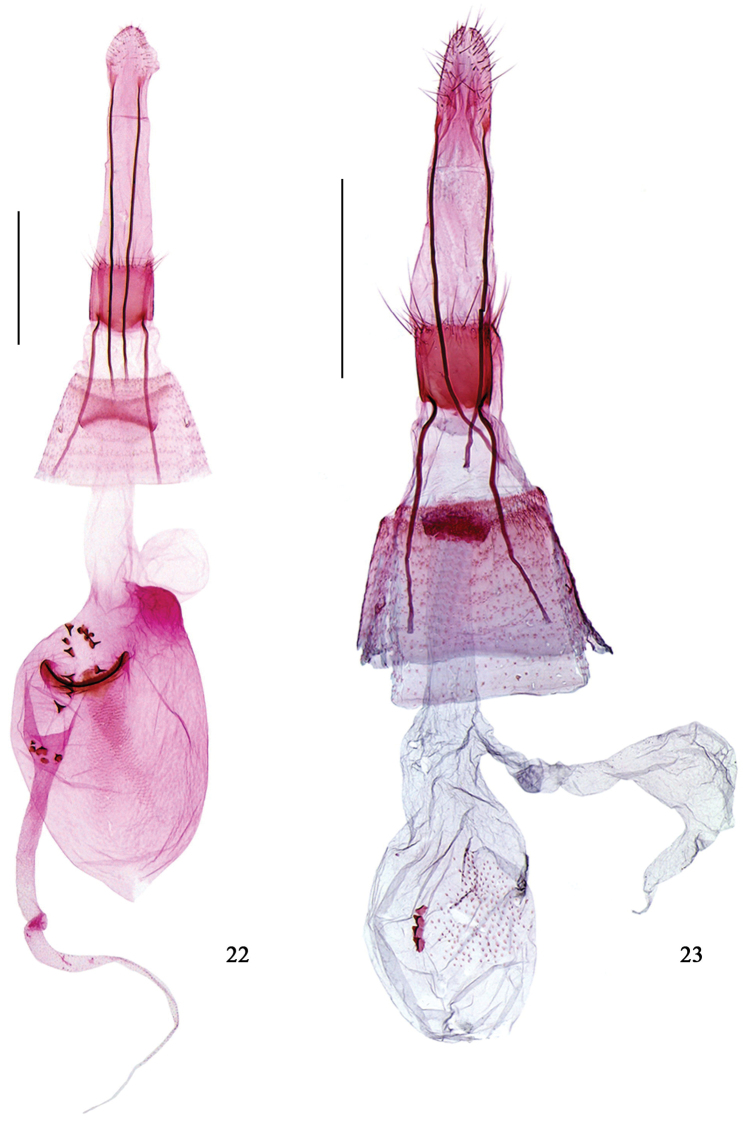
Female genitalia of *Hieromantis* species. **22** *Hieromantis kurokoi* Yasuda, slide No. GW13141 **23** *Hieromantis sheni* Li & Wang, slide No. GW14030. Scale bars: 0.5 mm.

**Diagnosis.** Adult (Fig. 11) with wingspan 6.0−11.0 mm. This species is similar to *Hieromantis ephodophora* Meyrick, 1897 by having similar forewing markings, but *Hieromantis kurokoi* can be separated by the valva having a rounded cucullus in the male genitalia (Fig. 16); and the appendix bursae about the same length as the corpus bursae in the female genitalia (Fig. 22). In *Hieromantis ephodophora*, the cucullus is subtriangular, and the appendix bursae is about four times length of the corpus bursae (Kasy 1973: Figs 97–98).

#### Distribution.

China (Anhui, Chongqing, Fujian, Gansu, Guangxi, Hainan, Hebei, Henan, Hubei, Hunan, Jiangxi, Shaanxi, Shanxi, Tianjin, Zhejiang), Japan, Russia.

#### Host plant.

*Cuscuta japonica* Choisy (Convolvulaceae).

### Hieromantis sheni

Taxon classificationAnimaliaLepidopteraStathmopodidae

Li & Wang, 2002

Figs 12 , 17 , 23

Hieromantis sheni Li & Wang, 2002: 503. Type locality: China (Henan).

#### Type material.

**CHINA: Holotype** ♂, Huangshian Village, Xixia County, Henan Province, 890 m, 17.vii.1998, leg. Houhun Li. **Paratypes: Henan Province**: 2♀, Mt. Jigong, Xinyang City, 700 m, 9.v.1997, leg. Houhun Li; 12♂, 5♀, Mt. Song, Dengfeng City, 800 m, 9.vi.2000, leg. Haili Yu; **Hebei Province**: 2♂, 3♀, Mt. Xiantai, Jingxing County, 1200 m, 23−24.vi.2000, leg. Haili Yu; **Jiangxi Province**: 1♀, Yushan County, 19.vi.1985, leg. Guangpu Shen.

#### Additional material.

**CHINA: Tianjin**: 1♀, Heishuihe, Mt. Baxian, Ji County, 550 m, 11.viii.2009, leg. Jing Zhang *et al.*; 3♂, 2♀, Heishuihe, Mt. Baxian, Ji County, 550 m, 2−14.viii.2010, leg. Yinghui Mou & Shurong Liu; **Hebei Province**: 1♂, Mt. Xiantai, Jingxing County, 1200 m, 24.vii.2000, leg. Haili Yu; **Shanxi Province**: 1♀, Dahe Forest Farm, 1340 m, 12.vii.2012, leg. Qiang Gao & Na Chen; 1♂, 1♀, Dashuang Village, Magedang Town, Lingchuan County, 773 m, 22.vii.2012, leg. Wei Guan & Xiuchun Wang; **Zhejiang Province**: 1♀, Mt. Fengyang, Longquan County, Lishui City, 1470 m, 31.vii.2007, leg. Qing Jin; 3♀, Sanmuping, Mt. Tianmu, 789 m, 15.vii.2014, leg. Aihui Yin, Xuemei Hu & Qingyun Wang; **Henan Province**: 2♀, Mt. Baiyun, Luoyang City, 1560 m, 21.vii.2001, leg. Dandan Zhang; 7♂, 13♀, Shaolin Temple, Mt. Song, Dengfeng City, 700 m, 15−17.vii.2002, leg. Xinpu Wang; 8♂, 5♀, Mt. Baiyun, Song County, 1580 m, 18−23.vii.2002, leg. Xinpu Wang; 1♀, Zhuyu Peak, Mt. Yuntai, Jiaozuo City, 1297 m, 5.viii.2014, leg. Peixin Cong, Sha Hu & Linjie Liu; **Hubei Province**: 1♀, Songbai Town, Shennongjia Forest, 1400 m, 17.vii.2003, leg. Shulian Hao; **Chongqing**: 2♀, Laosimianshan Village, Mt. Simian, 14.vii.2012, leg. Yinghui Sun & Aihui Yin; 2♀, Laosimianshan Village, Mt. Simian, 1280 m, 10.viii.2012, leg. Xiaofei Yang & Tengteng Liu; **Yunnan Province**: 1♀, Taiyanghe Nature Reserves, 1450 m, 30.vii.2014, leg. Zhenguo Zhang; **Shaanxi Province**: 7♂, 3♀, Haoping Temple, Mt. Taibai, 13−16.vii.2005, leg. Ping You.

#### Diagnosis.

Adult (Fig. 12) with wingspan 7.0−10.5 mm. This species is similar to *Hieromantis makiosana* Yasuda, 1988 by having an obliquely upward-oriented triangular cucullus, but can be separated by having a leaden gray thorax and the distal 1/4 of the forewing dark brown; and the uncus truncate apically in the male genitalia (Fig. 17); the presence of a signum and the appendix bursae about the same length as the corpus bursae in the female genitalia (Fig. 23). In *Hieromantis makiosana*, the thorax is white, and the forewing is ochreous yellow in the distal 1/5; the uncus is pointed at apex; the corpus bursae lacks a signum, and the appendix bursae is longer than four times length of the corpus bursae. This species is also similar to *Hieromantis phaeodora* Meyrick, 1929, and the differences between them are stated under the latter species.

#### Distribution.

China (Chongqing, Hebei, Henan, Hubei, Jiangxi, Shaanxi, Shanxi, Tianjin, Yunnan, Zhejiang).

## Supplementary Material

XML Treatment for Hieromantis

XML Treatment for Hieromantis rectangula

XML Treatment for Hieromantis arcuata

XML Treatment for Hieromantis puerensis

XML Treatment for Hieromantis phaeodora

XML Treatment for Hieromantis kurokoi

XML Treatment for Hieromantis sheni
